# Epidemiological Patterns of Gastrointestinal Parasitic Infections in Equine Populations from Urumqi and Ili, Xinjiang, China

**DOI:** 10.3390/vetsci12070644

**Published:** 2025-07-06

**Authors:** Yabin Lu, Penghui Ru, Sinan Qin, Yukun Zhang, Enning Fu, Mingyue Cai, Nuermaimaiti Tuohuti, Hui Wu, Yi Zhang, Yang Zhang

**Affiliations:** 1 College of Veterinary Medicine, Xinjiang Agricultural University, Urumqi 830052, China; lyb4095@163.com (Y.L.); 220220678@stu.xjau.edu.cn (P.R.); 320232806@xjau.edu.cn (S.Q.); 220220711@stu.xjau.edu.cn (Y.Z.); 220230843@stu.xjau.edu.cn (E.F.); 220220766@stu.xjau.edu.cn (M.C.); 320242878@xjau.edu.cn (N.T.); 2 Xinjiang Uygur Autonomous Region Aquaculture and Fisheries Development Center, Urumqi 830000, China; 18290631705@163.com

**Keywords:** equine gastrointestinal nematodes, strongylidae prevalence, pasture vs. stable management, Xinjiang equine parasitology, *Parascaris equorum*, *Eimeria*

## Abstract

Gastrointestinal parasitic diseases pose significant health risks to equine populations. This study investigated the epidemiological patterns of equine gastrointestinal parasites in Xinjiang by analyzing 83 fecal samples collected from Ili (*n* = 62) and Urumqi (*n* = 21) between August and November 2024. The modified McMaster technique was employed to quantify fecal egg counts (EPG) and was complemented by morphological identification to assess infection dynamics related to geography, breed specificity, and management practices. The results demonstrated an overall infection prevalence of 66.3% (55/83), with strongyles, *Parascaris equorum*, and *Eimeria* oocysts being present. Significant geographical variation was observed, with Ili exhibiting a higher prevalence (74.2%) compared to Urumqi (42.9%). Breed susceptibility analysis revealed that there was a 94.1% prevalence in Yili horses versus 42.9% in Kazakh horses. Pasture-managed herds showed markedly higher infection rates (94.1%) than stable-based systems (50.0%). Parasite community composition was dominated by strongyles (82.1%), followed by *Triodontophorus* spp. (27.7%) and *P. equorum* (2.4%). These findings highlight severe parasitic infection risks in Xinjiang’s grazing equids, underscoring the urgency of implementing targeted anthelmintic protocols to mitigate disease transmission.

## Simple Summary

Intestinal parasites are a major health concern for horses, particularly in grazing regions. This study examined parasite infections in horses from two areas in Xinjiang, China—Ili and Urumqi—to understand how location, horse breed, and feeding practices affect infection risks. The researchers collected 83 horse stool samples between August and November 2024, subsequently testing them in a lab to count parasite eggs and identify the types of endoparasites present. The results showed that 66% of horses were infected, with higher rates in those from Ili (74%) compared to those from Urumqi (43%). Yili horses had a much higher rate of infection (94%) than Kazakh horses (43%), and grazing horses were far more likely to carry parasites (94%) than those kept in stables (50%). The most common parasites were small strongyles, which were found in 82% of infected horses. These findings highlight the urgent need for tailored deworming plans, especially for grazing horses, to reduce infections and protect horse health. This work can help farmers and veterinarians prioritize parasite control strategies in high-risk areas, thus improving animal welfare and supporting sustainable horse farming.

## 1. Introduction

China has practiced equine husbandry for a long time, with the equine industry representing a key part of its livestock sector [1]. Historically, horses served as critical draft animals and transportation assets, contributing substantially to agricultural productivity and rural economic development. With socioeconomic advancement, the traditional roles of horses in labor and transportation have gradually been replaced, thus giving rise to the modern equine industry. Industry analyses indicate that the entire equine industry chain generates approximately CNY 70 billion (USD 9.7 billion) in output value. As of 2023, data revealed that the equine population in Xinjiang Uygur Autonomous Region, China, reached 1.134 million [2]. However, infectious diseases pose serious threats to the sustainable development of the equine industry, particularly parasitic infections, of which gastrointestinal nematode infections show the highest prevalence [3,4]. Despite Xinjiang’s position as the national core area for horse industry development, systematic research on equine gastrointestinal parasites remains insufficient. In-depth studies in this field will not only promote healthy industry development but will also represent an important practice in implementing animal welfare principles.

Gastrointestinal nematodes represent one of the most prevalent parasitic groups infecting equine species. The primary damage they inflict manifests through inducing significant pathological changes; disrupting gastrointestinal functions; and impairing the body condition, health, reproductive capacity, and lifespan of horses [5]. Studies confirm that 6.63% of foal mortality shows a direct correlation with gastrointestinal nematode infections [6]. The gastrointestinal parasites affecting equines encompass diverse species, predominantly including *Strongylus equinus*, *S. edentatus*, *S. vulgaris*, *Triodontophorus*, *Craterostomum*, and *Oesophagodontus* from the Strongylidae family; *Trichonema*, *Poteriostomum*, *Gyalocephalus*, and *Cylicocyclus* from the Trichonematidae family; and *Oxyuris equi* and *Parascaris equorum*. Although research indicates that over 40 nematode species can parasitize horses concurrently, only a few species dominate clinical infections [7,8,9,10]. Among these, *S. equinus*, *S. edentatus*, *S. vulgaris*, and *P. equorum* pose the most severe threats to equine health. The first three of these species, collectively termed large strongyles, cause mesenteric artery embolism, intestinal colic, and necrotic intestinal wall lesions through larval migration in the digestive tract [11,12]. *P. equorum* larvae induce interstitial hepatitis and focal pneumonia during hepatopulmonary migration, while heavy adult infestations may lead to intestinal obstruction, perforation, and mortality in foals [13]. Although other *Strongylidae* species lack tissue migration capacity, they demonstrate high parasitic loads in equines, thus constituting the principal components of gastrointestinal nematode communities [7]. These parasites can cause progressive weight loss, chronic enteritis, and other clinical manifestations.

Currently, the diagnostic methods used to combat equine gastrointestinal parasites mainly include novel ones, such as enzyme-linked immunosorbent assays (ELISAs), polymerase chain reaction (PCR), and DNA metabarcoding assays, as well as traditional pathogen diagnostic methods, such as fecal egg count (FEC) [14,15]. ELISAs can detect latent infections and are suitable for large-scale screening, yet they cannot differentiate between current and past infections in animals [14]. PCR detects parasitic DNA in fecal samples by amplifying species-specific genetic fragments [16], thus offering high specificity and sensitivity. Fluorescence-based quantitative PCR (qPCR), an enhanced version of this technique, provides even greater sensitivity and specificity [17]. However, ELISA, conventional PCR, and qPCR typically target only a limited number of pathogens per test, thus limiting their capacity to comprehensively assess the overall parasitic infection profile in equine gastrointestinal systems [15]. The DNA metabarcoding assay employs the PCR amplification of specific DNA regions followed by high-throughput parallel sequencing to analyze complex biological communities, and it has been widely applied in microbial community studies and environmental biodiversity monitoring [15,18,19,20]. Its key advantage lies in its simultaneous, high-throughput detection of multiple parasite species within a single fecal sample, thus enabling a more holistic understanding of parasitic species composition and distribution in horses [15]. Nevertheless, this approach involves high costs and a lengthy workflow—spanning sample preparation, quality control, PCR amplification, and sequencing—which typically requires nearly one month to complete, thus rendering it impractical for clinical diagnostics. FEC has remained a cornerstone of parasitological diagnostics in veterinary parasitology for over a century, maintaining its central role in both parasitological research and clinical diagnosis [21]. However, this method cannot reliably differentiate strongyles due to the morphological similarity of their eggs. Over the past decade, an increasing number of countries have emphasized the importance of routine FEC implementation, primarily owing to the following advantages: First, it enables the quantification of parasitic infection intensity, allowing adult horses to be categorized into low-, moderate-, or high-intensity infection groups for prevention and control strategies. Second, FEC serves as an effective tool for evaluating the efficacy of anthelmintic treatments. Finally, it allows for critical surveillance for strongyles and *P. equorum* in foals [22,23].

Xinjiang constitutes a hyperendemic region for equine gastrointestinal nematodes in China. Historical surveillance data indicate that there was an 89.1% overall infection rate of gastrointestinal parasites in Ili Valley horses in 2012 [24], with the subsequent monitoring in Zhaosu County (2014) demonstrating a sustained prevalence exceeding 80% [25]. Nevertheless, empirical data remain scarce regarding the influencing mechanisms of husbandry management systems, geographical populations, and genetic lineages on nematode infection patterns. Conducting systematic epidemiological investigations not only constitutes a foundation for developing scientific control strategies but also represents a critical balance between parasitic disease management and mitigating anthelmintic resistance risks [6].

Therefore, this study aims to establish a targeted parasite control framework through a comparative analysis of gastrointestinal nematode infections at two Xinjiang sampling sites (Ili and Urumqi), specifically seeking to identify predominant parasitic nematode species, quantify equine gastrointestinal parasite infection burden, and provide theoretical foundations for developing precision-based helminth control strategies.

## 2. Materials and Methods

### 2.1. Sample Collection

The sampling protocol for this study was approved by farm owners and did not interfere with normal farm operations. A total of 83 fecal samples were collected. On 15 August 2024, 62 samples were collected from a large equine facility in Ili, Xinjiang, which maintained a herd size of 1200 horses (sampling proportion: 5%). Between 5 and 6 November 2024, 21 additional samples were obtained from two household farms near Urumqi, Xinjiang: 6 samples (40% sampling rate) from Farm 1 (15-horse herd) and 15 samples (43% sampling rate) from Farm 2 (35-horse herd). The study population comprised 21 pasture-fed Kazakh horses and 62 Yili horses (28 stable-fed and 34 pasture-fed). All subjects had no history of prophylactic deworming interventions. All specimens were collected immediately after defecation, with the unoxidized surface layer selected for sampling. Each sample was individually packaged using a “one-horse-one-container” protocol, with specimen information being directly indicated on container surfaces. To prevent cross-contamination, disposable gloves were replaced between each sample collection process. The samples underwent immediate preservation in 4 °C portable refrigerators within 30 min post-collection. All specimens were transported on ice to the Parasitology Laboratory at the College of Veterinary Medicine, Xinjiang Agricultural University, on the day of collection.

### 2.2. Fecal Egg Counts

Fecal egg counts (FECs) were completed within one week of sampling using a modified McMaster technique. Briefly, 2 g of feces was homogenized with 10 mL of saturated saline solution in a mortar, followed by the addition of 50 mL of saturated saline. The mixture was filtered through a fecal sieve, and the filtrate was transferred into two standard McMaster counting chambers. After 5 min of sedimentation at room temperature, eggs were counted under a microscope. Each egg was photographed, and its length was measured. Parasite infection intensity was assessed using the following classification criteria: <200 eggs per gram (EPG) or oocysts per gram (OPG) of feces indicating low-intensity infection, 200–500 EPG or OPG indicating moderate-intensity infection, and ≥500 EPG or OPG indicating high-intensity infection [5,26,27,28].

During counting, all strongyle eggs were initially included in the total count, then subclassified as follows based on morphological criteria: small strongyle eggs (<90 μm), large strongyle eggs (≥90 μm), and *Triodontophorus* spp. (≥120 μm) [5]. *P. equorum* eggs and *Eimeria* oocysts were enumerated separately [29,30]. EPG and OPG were calculated as the mean count from both chambers multiplied by 200.

### 2.3. Statistical Analyses

Subsequently, 95% confidence intervals (CIs) were calculated for positive rates via VasarStats (http://vassarstats.net/index.html) (accessed on 20 May 2025) to estimate the potential ranges of true prevalence. SPSS 27 (SPSS Software, IBM, Chicago, USA) was used to calculate *p*-Values. For the comparative analysis of infection rate differences between breeds, this study established comparative groups comprising 21 fecal samples from Kazakh horses in Urumqi and 34 samples from Yili horses in Ili, with both groups maintained under pasture-based management to control for husbandry variables. When evaluating management system influences, 62 Yili horses of identical breed were selected as study subjects: 28 under stable-based management and 34 pasture-managed, thus ensuring breed homogeneity to eliminate genetic background interference.

## 3. Results

### 3.1. Descriptive Data Analysis

**Figure 1:**
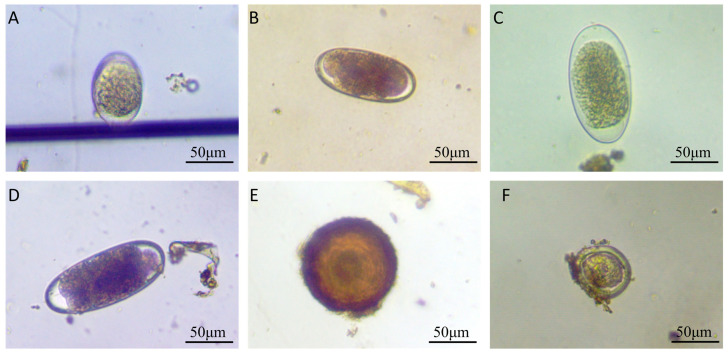
Morphological characteristics of gastrointestinal parasite eggs or oocysts in horse feces. (**A**) Strongyle egg (88 μm × 56 μm); (**B**) strongyle egg (109 μm × 50 μm); (**C**) *Triodontophorus*-like egg (124 μm × 62 μm); (**D**) *Triodontophorus*-like egg (132 μm × 56 μm); (**E**) *Parascaris equorum* egg (112 μm × 109 μm); (**F**) *Eimeria* spp. oocyst (62 μm × 59 μm).

A total of 83 equine fecal samples were collected, with 21 being from Urumqi and 62 being from Ili. Microscopic examination revealed parasite eggs/oocysts in 55 samples, yielding an overall infection rate of 66.3% (95% CI: 55.0–76.1%). A total of 9 positive samples were identified in Urumqi, compared to 46 in Ili (Table 1 and Table S1). Chi-square analysis demonstrated a significant difference in infection rates between the two regions (*p* = 0.009). Kazakh horses demonstrated a 42.9% (9/21) gastrointestinal parasite prevalence, which is markedly lower than the 94.1% (32/34) observed in Yili horses (*p* < 0.001). Management system comparisons showed that stable-based horses had 50.0% infection prevalence (14/28), which is significantly lower than the 94.1% seen in pasture-based systems (*p* < 0.001). Among the 55 positive samples, the predominant intestinal parasite was strongyles, and *P. equorum* and *Eimeria* spp. were also found (Figure 1).

**Figure 2:**
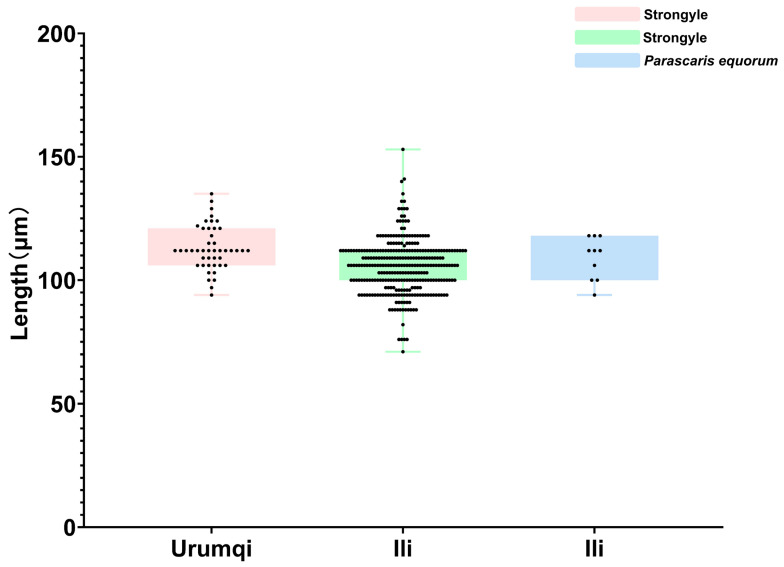
Length measurements of helminth eggs.

**Figure 3:**
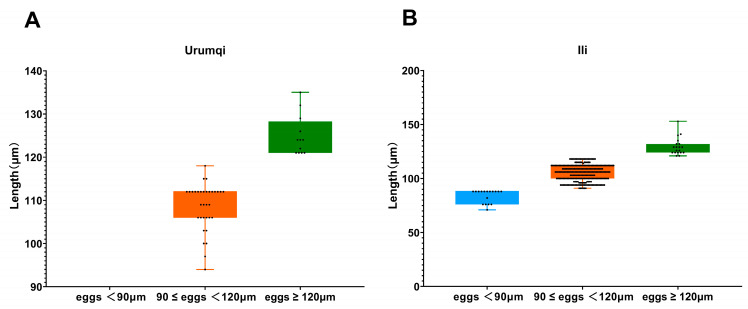
Length measurements of strongyles eggs across different areas. (**A**) Egg length distribution of strongyles in Urumqi; (**B**) egg length distribution of strongyles in Ili.

While measuring the length of strongyle eggs, it was found that 17 eggs measured below 90 μm, with the shortest being 71 μm and the longest being 88 μm; the mean length was 83.8 μm. A total of 299 eggs measured between 90 and 120 μm, ranging from 91 μm (shortest) to 118 μm (longest), with a mean length of 105.6 μm. Meanwhile, 31 eggs exceeded 120 μm and were identified as *Triodontophorus* spp., measuring between 121 μm (shortest) and 153 μm (longest), averaging 127.8 μm in length. Among the 10 detected *P. equorum* eggs, a maximum length of 118 μm, minimum length of 94 μm, and mean length of 109 μm were seen. In the three identified *Eimeria* oocysts, the measurements showed a maximum length of 62 μm, minimum length of 47 μm, and mean length of 53 μm (Figure 2 and Figure 3).

### 3.2. Infection Intensity of Different Parasite Species

Among the 83 samples, the detection rate of strongyle eggs was 65.1% (54/83), with a fecal egg count range from 100 to 2300 EPG and an overall average of 643 EPG. In the Urumqi and Ili regions, the detection rates of strongyles eggs were 42.9% (9/21) and 72.6% (45/62), with average fecal egg counts of 500 and 671 EPG, respectively. Eleven samples contained strongyle eggs smaller than 90 μm, resulting in a total infection rate of 13.3% (11/83), and these were only found in Ili, with an average egg count of 155 EPG. The highest infection rate was observed in samples with strongyle egg lengths between 90 and 120 μm, with a total infection rate of 59.0% (50/83) and an average egg count of 598 EPG. In Urumqi, the infection rate was 28.6% (6/21), with an average egg count of 550 EPG, while in the Ili region, the infection rate was 71.0% (44/62), with an average egg count of 605 EPG. The total infection rate of *Triodontophorus* spp. was 26.5% (22/83). In Urumqi, the infection rate was 28.6% (6/21), with an average egg count of 200 EPG, while in Ili, the infection rate was 25.8% (16/62), with an average egg count of 119 EPG. Only two samples were infected with *P. equorum*, resulting in an infection rate of 2.4% (2/83), with an average egg count of 500 EPG, and these were also only found in Ili. Three samples were infected with *Eimeria* spp., resulting in an infection rate of 3.6% (3/83), with an average oocyst count of 100 OPG (Table 2 and Table S1).

**Figure 4:**
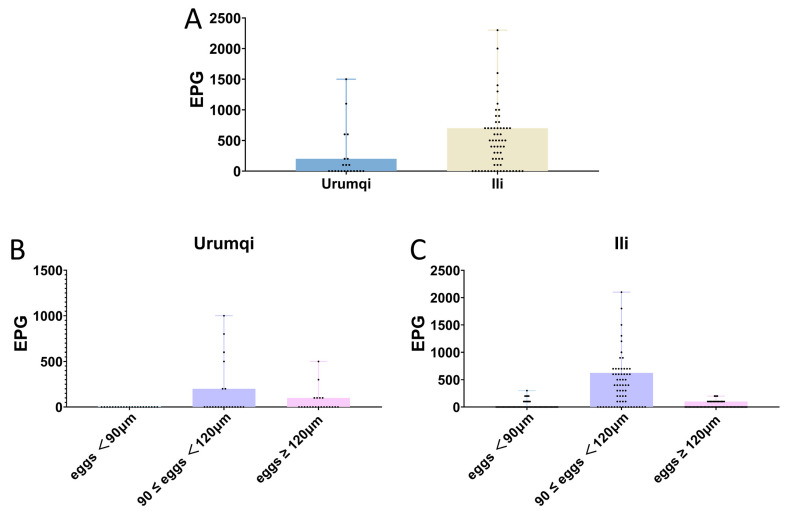
Regional variations in parasite infection intensity. (**A**) Comparative analysis of parasite infection intensity between Urumqi and Ili. (**B**) Strongyle infection intensity in Urumqi. (**C**) Strongyle infection intensity in Ili.

According to the strongyle infection intensity assessment criteria, among the nine infected samples from Urumqi, three were classified as low-intensity infections, two as moderate-intensity infections, and the remaining four were classified as high-intensity infections. Of the 45 infected samples from Ili, 3 were low-intensity infections, 18 were moderate-intensity infections, and 24 were high-intensity infections. Samples with egg lengths below 90 μm all exhibited low-to-moderate infection intensities. For samples with egg lengths between 90 and 120 μm, the infection intensity distribution was as follows: 4 low-intensity cases, 21 moderate-intensity cases, and 25 high-intensity cases. In the *Triodontophorus* spp. group, 17 were classified as low-intensity infections and the remaining 5 as moderate-intensity infections, while no high-intensity infections were detected (Figure 4).

## 4. Discussion

This study investigated gastrointestinal parasites in horses from two regions of Xinjiang, revealing a total infection rate of 66.3%, which is comparable to the rates reported in Ethiopia (56.6%) [31], Australia (62%) [32], and Thailand (74.7%) [33]. Notably, 28 fecal samples exhibited high-intensity infections (≥500 EPG). The high infection rates and intensities observed suggest that Xinjiang’s unique climatic and environmental conditions strongly favor the transmission of gastrointestinal parasites in equids. Consequently, this underscores the critical necessity of establishing a comprehensive prevention and control system for equine parasitic diseases in the region.

Owing to its high specificity and sensitivity, this study primarily employed FEC for the detection of gastrointestinal parasites [23]. Additionally, given the scarcity of reports on equine gastrointestinal parasite infections in Xinjiang over the past five years, there was an urgent need to comprehensively understand the current status of such infections. FEC not only enables the clear identification of the types of gastrointestinal parasites present in horses but also provides insights into infection intensity. During the preliminary screening of detection methods, we attempted to extract DNA from fecal samples and performed PCR amplification using nematode-specific primers. However, electrophoresis revealed substantial non-specific amplification bands, which precluded reliable species identification through sequencing (unpublished data). Consequently, in alignment with the objectives of this study, we ultimately opted for FEC.

The parasite spectrum identified in this study (strongyles, *P. equorum*, *Eimeria* spp.) corresponds with historical reports from northwest China [24]. Furthermore, after measuring the length of each egg, we categorized strongyle eggs into three groups based on length discrepancies: eggs < 90 μm, eggs ranging from 90 to 120 μm, and eggs > 120 μm (classified as *Triodontophorus* spp.). The results demonstrated that eggs measuring 90–120 μm in length dominated both infection rates and infection intensity in this investigation. According to Kuzmina’s measurements of *S. vulgaris* and *S. edentatus* eggs, the mean length of *S. vulgaris* eggs was 80.46 μm, while *S. edentatus* eggs averaged 79.80 μm [34]. Therefore, we defined eggs < 90 μm as large strongyle eggs and those measuring 90–120 μm as small strongyle eggs. In the current study, only 11 samples contained eggs smaller than 90 μm, and all were collected from Ili. Notably, samples with strongyle eggs < 90 μm exhibited consistently low EPG values, averaging 155 EPG, which is indicative of mild-to-moderate infection intensity. This pattern leads us to infer that the prevalence of large strongyle infections in Urumqi may have been effectively controlled through prolonged anthelmintic interventions—a hypothesis supported by evidence from other countries. For instance, since 1960, the recommended approach to controlling equine nematodes has been to administer anthelmintic treatment to all horses every 6–8 weeks, which significantly reduces the prevalence of common Strongylus infections [6]. Sweden achieved a dramatic reduction in *Strongylus vulgaris* prevalence from 40 to 60% (1979) to 5% (1990) through systematic deworming programs [35].

Based on the findings of our investigation, defining eggs < 90 μm as large strongyles eggs allows us to conclude that, even without anthelmintic use, the infection rate of large strongyles was effectively controlled. However, the infection intensity of small strongyle eggs (90–120 μm) remains a significant concern. An analysis of infection intensity via EPG revealed 25 samples with heavy small strongyle infections, accounting for 50% of all infections. This suggests potential emerging anthelmintic resistance in small strongyle populations across both sampling sites. Notably, this issue is not confined to Xinjiang, China; Australia reports small strongyle infection rates as high as 72–100% [28]. Consequently, future control strategies should prioritize surveillance and intervention targeting small strongyle species.

Geographical environmental disparities significantly influence the infection rates of gastrointestinal parasites in horses. The infection rate in Urumqi was 42.9%, compared to 74.2% in Ili. Although no anthelmintic treatments were administered at either site, infection rates were significantly higher in Ili than in Urumqi. Elevated precipitation levels, along with higher temperatures and humidity, were identified as direct contributors to the increased prevalence of parasitic infections [36]. Compared to Urumqi, Ili indeed has a significantly warmer climate with substantially higher rainfall and humidity levels, which correlates with the more severe prevalence of gastrointestinal parasitic infections in equine populations within the region.

An analysis of inter-breed variations revealed a highly significant difference in gastrointestinal parasite infection rates between Yili horses and Kazakh horses. Cross-species comparisons suggest potential interspecific differences in parasitic susceptibility among equids (horses/donkeys/mules), as evidenced by the 2014 finding of an 82% infection rate in horses in Zhaosu County [25], compared to a contemporaneous 60% infection rate in donkeys in southern Xinjiang [37]. However, it is important to note that current data have not controlled for confounding variables, such as geographical region and husbandry practices, and these conclusions require validation through multifactorial controlled experiments.

Extensive data indicate a significant association between grazing and strongyles infection [35,38,39,40]. This study confirms that the infection rate in grazing horses (94.1%) is significantly higher than that in stabled horses. Compared to the 2012 data from the Ili River Valley (89.1%) [24], our findings reveal a continuing upward trend in infection rates among grazing horses. Notably, among the 11 samples containing strongyles eggs <90 μm (classified as large strongyles), only 1 sample originated from stabled horses, while the remaining 10 were all derived from grazing horses. This further demonstrates that suspected large strongyle infection rates are also significantly higher in grazing horses compared to stabled horses. Based on these results, we recommend that grazing horses should adhere to regular anthelmintic treatment protocols to reduce infection prevalence.

The infection rate of *P. equorum* has declined significantly. In 2012, the prevalence of *P. equorum* in grazing horses in Ili was 38.0% [24], whereas this survey detected equine ascarid eggs in only two samples from Ili’s grazing horse population. However, data from 2019 to 2021 indicate that donkey populations in southern Xinjiang maintained infection rates exceeding 40% [41]. This decline may reflect the successful control of *P. equorum* in both regions, though continued surveillance is necessary to confirm long-term efficacy.

In this study, coccidian oocysts were detected in three samples, yielding an infection rate of 3.6%, which is relatively close to the prevalence reported in Iran (0.88%) [42]. Based on oocyst morphology and dimensions, these were preliminarily identified as *Eimeria leuckarti*. This pathogen can cause clinical manifestations such as diarrhea and emaciation in foals, with severe infections potentially leading to mortality.

This study also has several limitations. A notably prominent limitation is the relatively small sample size, particularly in Urumqi, which compromised the precision of assessment regarding the true prevalence of equine gastrointestinal parasites in this region. Therefore, future research should expand the sampling scope and sample size, while incorporating additional influencing factors, such as different seasons and breeding management practices, to comprehensively evaluate the prevalence of gastrointestinal parasites in Xinjiang, China.

## 5. Conclusions

In conclusion, our findings demonstrate that small strongyles remain as highly prevalent gastrointestinal parasites in both the Urumqi and Ili regions. Additionally, pastured horses exhibited exceptionally high parasite infection rates. Future control strategies should prioritize surveillance and intervention, specifically targeting grazing horse populations, with a particular emphasis on the management of small strongyle infections.

## Figures and Tables

**Table 1:** Prevalence of gastrointestinal parasites found in 83 horses.

Variable		Positive	Total	Prevalence (%)	95% Confidence Interval	*p*-Value
Region	Urumqi	9	21	42.9	22.6–65.6	<0.01
	Ili	46	62	74.2	61.3–84.1	
Breed	Kazak horse	9	21	42.9	22.6–65.6	<0.001
	Yili horse	32	34	94.1	79.0–99.0	
Management system	Stable	14	28	50.0	31.1–68.9	<0.001
	Pasture	32	34	94.1	79.0–99.0	
	Total	55	83	66.3	55.0–76.1	

**Table 2:** Details of strongyles, *Parascaris equorum*, and *Eimeria* spp. fecal egg/oocyst counts in different regions.

Site	Positive Value of Total Strongyles (EPG)	Positive Value of Strongyles of Eggs < 90 μm (EPG)	Positive Value of 90 μm ≤ Strongyles of Eggs < 120 μm (EPG)	Positive Value of *Triodontophorus* spp. (EPG)	Positive Value of *P. equorum* (EPG)	Positive Value of *Eimeria* (OPG)
Urumqi	9 (100–1500)	0	6 (200–1000)	6 (100–500)	0	0
Ili	45 (100–2300)	11 (100–300)	44 (100–2100)	16 (100–200)	2 (100–900)	3 (100)
Total	54 (100–2300)	11 (100–300)	50 (100–2100)	22 (100–500)	2 (100–900)	3 (100)

## Data Availability

Data are contained within this article, and further inquiries can be directed to the corresponding authors.

## Supplementary Materials

The following supporting information can be downloaded at: https://www.mdpi.com/article/10.3390/vetsci12070644/s1, Table S1 Gastrointestinal parasite infection intensity in horses: regional, breed, and management-specific variations.
