# Supplementary material for: Epidemiological Patterns of Gastrointestinal Parasitic Infections in Equine Populations from Urumqi and Ili, Xinjiang, China
Source: Vet Sci. 2025 Jul 6;12(7):644. doi: 10.3390/vetsci12070644 (PMC12299244; doi:10.3390/vetsci12070644)
Supplement: Supplementary file 1 [file vetsci-12-00644-s001.zip › vetsci-3643923-supplementary.pdf]

Table S1. Gastrointestinal parasite infection intensity in horses: regional, breed, and management-specific variations

Number	Region	Breed	manage system	Positive Value of Strongyles (EPG)				Positive Value of *P. equorum* (EPG)	Positive Value of *Eimeria* (OPG)
				Total Strongyles	Strongyles of Eggs < 90μm	90μm≤Strongyles of Eggs < 120μm	*Triodontophorus* spp.		
1	Urumqi	Kazak horse	Pasture	100	0	0	100	0	0
2				1500	0	1000	500	0	0
3				200	0	200	0	0	0
4				600	0	500	100	0	0
5				0	0	0	0	0	0
6				0	0	0	0	0	0
7				0	0	0	0	0	0
8				0	0	0	0	0	0
9				0	0	0	0	0	0
10				0	0	0	0	0	0
11				0	0	0	0	0	0
12				0	0	0	0	0	0
13				0	0	0	0	0	0
14				0	0	0	0	0	0
15				100	0	0	100	0	0
16				0	0	0	0	0	0
17				100	0	0	100	0	0
18				0	0	0	0	0	0
19				200	0	200	0	0	0
20				1100	0	800	300	0	0
21				600	0	600	0	0	0
22	Ili	Yili horse	Stable	500	0	500	0	0	0
23				100	0	100	0	0	0
24				200	0	200	0	0	0
25				500	0	400	100	0	0
26				0	0	0	0	0	0
27				300	0	200	100	0	0
28				0	0	0	0	0	0
29				0	0	0	0	0	0
30				0	0	0	0	0	0
31				0	0	0	0	0	0
32				200	0	100	100	0	0
33				0	0	0	0	0	0
34				0	0	0	0	0	0
35				600	0	400	200	0	0
36				0	0	0	0	0	0
37				100	0	100	0	0	0
38				0	0	0	0	0	0
39				700	0	600	100	0	0
40				1000	0	900	100	0	0
41				0	0	0	0	0	0

42			0	0	0	0	0	0
43			0	0	0	0	0	100
44			700	100	600	0	0	0
45			600	0	600	0	0	0
46			200	0	200	0	0	0
47			0	0	0	0	0	0
48			0	0	0	0	0	0
49			0	0	0	0	0	0
50	Ili	Pasture	0	0	0	0	0	0
51			900	200	700	0	0	0
52			400	0	400	0	0	0
53			400	0	300	100	0	0
54			2000	0	1800	200	0	100
55			1600	0	1500	100	0	0
56			500	0	500	0	0	0
57			700	0	700	0	0	0
58			700	0	700	0	0	0
59			600	0	500	100	0	0
60			700	0	700	0	0	0
61			1300	0	1300	0	0	0
62			1000	0	1000	0	0	100
63			700	0	700	0	900	0
64			700	0	700	0	0	0
65			800	0	700	100	0	0
66			300	0	300	0	100	0
67			700	0	600	100	0	0
68			300	300	0	0	0	0
69			0	0	0	0	0	0
70			400	100	300	0	0	0
71			200	0	200	0	0	0
72			500	0	400	100	0	0
73			400	100	300	0	0	0
74			1100	200	900	0	0	0
75			400	0	400	0	0	0
76			2300	100	2100	100	0	0
77			800	0	600	200	0	0
78			500	100	400	0	0	0
79			1400	200	1200	0	0	0
80			700	100	600	0	0	0
81			900	200	600	100	0	0
82			500	0	500	0	0	0
83			100	0	100	0	0	0
